# Neptune and His Lobsters

**Published:** 1910-11

**Authors:** J. W. Hurty

**Affiliations:** Secretary, Indianapolis


### Neptune and His Lobsters.

One time Neptune called his law-makers together to make laws and to provide means for carrying on his government. Before the end of the first week of the session it was plain that of all the different inhabitants of old Nep's realm principally one class was represented. How it came about no one could explain, but the proof was indubitable, mostly lobsters had been elected, only a few cuttle fish and two or three sharks had been thrown in. Well, there they were, the voters of the great deep had sent them up, and what could Nep do but accept them. Finally, the majority party of one branch of the law-makers selected one of their number, a lobster whose right claw had been torn off, to tell them when to get on and where to get off. This leader was a lobster all right, for he didn't know himself when he was on and when he was off. However, others observed he was quite always off. One old gray lobster said: "That head lobster with one claw gone hasn't sense enough to follow, let alone to lead." The fact was, the leader lobster kept backing all the time, and having only one claw, he backed in a circle. But going backward was natural to the whole bunch, and every one was satisfied.

As the days went by many economic and broad laws were proposed, but as they would benefit the whole kingdom, they could not, therefore, be considered. Finally, what was known as the rolley-polley bill was introduced, and then the skeedoozal bill. As both were clearly opposed to the best interests of the realm, the lobsters supported them heartily. The rolley-polley bill required that cuttle fish should hereafter be useful and secrete booze instead of ink; and the skeedoozal bill required that the sea cows should wear horns like land cattle. The cuttle fish and sea cows protested the proposed laws were contrary to nature, but the lobsters wouldn't listen, they passed them anyhow. You ask why? Because they were lobsters.—*Bulletin Indiana State Board of Health, Dr. J. W. Hurty, Secretary,* Indianapolis.

